# Typhoid Vaccine Acceleration Consortium Malawi: A Phase III, Randomized, Double-blind, Controlled Trial of the Clinical Efficacy of Typhoid Conjugate Vaccine Among Children in Blantyre, Malawi

**DOI:** 10.1093/cid/ciy1103

**Published:** 2019-03-07

**Authors:** James E Meiring, Matthew B Laurens, Pratiksha Patel, Priyanka Patel, Theresa Misiri, Kenneth Simiyu, Felistas Mwakiseghile, J Kathleen Tracy, Clemens Masesa, Yuanyuan Liang, Marc Henrion, Elizabeth Rotrosen, Markus Gmeiner, Robert Heyderman, Karen Kotloff, Melita A Gordon, Kathleen M Neuzil

**Affiliations:** 1 Oxford Vaccine Group, Department of Paediatrics, Oxford University, United Kingdom; 2 Malawi-Liverpool-Wellcome Trust Clinical Research Programme, Blantyre, Malawi; 3 Center for Vaccine Development and Global Health at the University of Maryland School of Medicine, Baltimore, MD; 4 Division of Infection and Immunity, University College London, United Kingdom; 5 Institute of Infection and Global Health, University of Liverpool, United Kingdom

**Keywords:** typhoid conjugate vaccine, Malawi, Africa, children/pediatric, TyVAC

## Abstract

**Background:**

Typhoid fever is an acute infection characterized by prolonged fever following the ingestion and subsequent invasion of *Salmonella enterica* serovar Typhi (*S.* Typhi), a human-restricted pathogen. The incidence of typhoid fever has been most reported in children 5–15 years of age, but is increasingly recognized in children younger than 5 years old. There has been a recent expansion of multidrug-resistant typhoid fever globally. Prior typhoid vaccines were not suitable for use in the youngest children in countries with a high burden of disease. This study aims to determine the efficacy of a typhoid conjugate vaccine (TCV) that was recently prequalified by the World Health Organization, by testing it in children 9 months through 12 years of age in Blantyre, Malawi.

**Methods:**

In this Phase III, individually randomized, controlled, double-blind trial of the clinical efficacy of TCV, 28 000 children 9 months through 12 years of age will be enrolled and randomized in a 1:1 ratio to receive either Vi-TCV or a meningococcal serogroup A conjugate vaccine. A subset of 600 of these children will be further enrolled in an immunogenicity and reactogenicity sub-study to evaluate the safety profile and immune response elicited by Vi-TCV. Recruiting began in February 2018.

**Results:**

All children will be under passive surveillance for at least 2 years to determine the primary outcome, which is blood culture–confirmed *S.* Typhi illness. Children enrolled in the immunogenicity and reactogenicity sub-study will have blood drawn before vaccination and at 2 timepoints after vaccination to measure their immune response to vaccination. They will also be followed actively for adverse events and serious adverse events.

**Conclusions:**

The introduction of a single-dose, efficacious typhoid vaccine into countries with high burden of disease or significant antimicrobial resistance could have a dramatic impact, protecting children from infection and reducing antimicrobial usage and associated health inequity in the world’s poorest places. This trial, the first of a TCV in Africa, seeks to demonstrate the impact and programmatic use of TCVs within an endemic setting.

**Clinical Trials Registration:**

NCT03299426.

Typhoid fever is an acute, systemic infection caused by the ingestion of the human restricted pathogen *Salmonella enterica* serovar Typhi (*S*. Typhi) [1]. Typhoid fever affects an estimated 12–27 million people globally each year, with 129 000–223 000 related deaths [2–5]. In endemic areas, the highest incidence of typhoid fever has been found in school-aged children (5–15 years) [6, 7], but typhoid is increasingly being recognized in children under 5 years of age [4, 5]. In countries with inadequate sanitation and water contamination, infections may be acquired from water and food contaminated with human waste [8]. In addition, typhoid may be transmitted via a food vehicle if handled by an individual who is a chronic carrier of *S.* Typhi [9].

The epidemiology of typhoid fever in Africa has been less well studied than in Asia. The recent Typhoid Fever Surveillance in Africa Program measured the incidence of invasive *Salmonella* bloodstream infections across 13 sites in 10 countries, with incidence estimates ranging from 0 to 383 per 100 000 in different sites. The incidence was highest in the 2–14-year age group and, importantly, 47% of *S*. Typhi isolates were multidrug resistant [10]. Updated incidence estimates following this study decreased in east Africa, to 348 per 100 000 people, but increased in west Africa, to 422 per 100 000 people [11]. Outbreaks of multidrug-resistant (MDR) typhoid fever have occurred throughout sub-Saharan Africa in recent years—including in Malawi, Uganda, Zimbabwe, Zambia, and the Democratic Republic of Congo—caused by strains that have been demonstrated to have originated in Asia. The rapid international spread of MDR typhoid is of great concern [12–16].

Starting in 1998, the Malawi Liverpool Wellcome Trust Clinical Research Programme has systematically collected blood and cerebrospinal fluid for culture from febrile patients at the Queen Elizabeth Central Hospital (QECH) in Blantyre, Malawi [17]. Between 1998 and 2010, there were approximately 14 cases of *S*. Typhi per year, with 6.8% demonstrating multidrug resistance. A rapid increase in cases began in 2011, peaking in 2014 with 782 cases and providing a minimum incidence estimate for Blantyre of 184/100 000 person-years of observation. These infections have resulted in a high mortality rate (2.5%), despite use of fluoroquinolone antibiotics [18, 19]. This increase is almost entirely due to the emergence and spread of the MDR H58 haplotype of *S*. Typhi. Mathematical modeling suggests the incidence will decline, then settle to a stable, endemic level [20, 21]. In Malawi, typhoid is isolated throughout the year, but does have a seasonal pattern, peaking at the end of the wet season and during the early dry season. Through the Strategic Typhoid Alliance Across Africa and Asia program, large, community-based studies are ongoing and provide estimates of disease burdens in the populations that will live in the site for this vaccine trial [22].

A new, first-generation typhoid Vi conjugate vaccine (Typbar-TCV) has been developed by Bharat Biotech International, Hyderabad, India. This vaccine consists of 25 μg of Vi polysaccharide, conjugated to a nontoxic tetanus toxoid protein carrier. Vi-TCV elicits a stronger anti-Vi response than unconjugated Vi polysaccharide, and the elicited antibodies have higher avidity than those detected after the unconjugated Vi vaccine [23]. The safety and immunogenicity of Vi-TCV has been studied in adults and children down to 6 months of age in India, and is highly immunogenic [24].

In the Oxford controlled human infection model, a single dose of Vi-TCV was well tolerated and demonstrated 54.6% protective efficacy in healthy adults when the primary endpoint of blood culture positivity or prolonged fever was reached. When a more comparative endpoint to real-world efficacy was used, of fever followed by bacteraemia, efficacy increased to 87%, which is comparable to previous field trials of other typhoid conjugate vaccines (TCVs) [25, 26].

In October 2017, the World Health Organization’s (WHO) Strategic Advisory Group of Experts (SAGE), in recognition of the high burden of typhoid and the increase in antimicrobial resistance in low- and middle-income countries, recommended the introduction of TCVs for children older than 6 months in typhoid-endemic countries [27]. Following the SAGE recommendation, Gavi announced an 85 million dollar window for 2019–2020 to support the introduction of TCVs in low-income countries [28]. In December 2017, the WHO approved the prequalification of Typbar-TCV, facilitating its introduction in low-income countries [29]. While these global policy and financing decisions are necessary for vaccine introduction in low-income countries, they are generally not sufficient for country-level uptake, particularly in Africa, where TCVs have never been tested. Malawi is a Gavi-eligible country, but has not submitted an application for support at the time of writing. The timeline from application to Gavi to Gavi acceptance and the country introduction of the vaccine is approximately 18 months. Trial results will, therefore, precede any other introduction, providing valuable efficacy data for the African continent.

In summary, the Typbar-TCV Vi conjugate vaccine is a promising candidate for the control of typhoid fever in Africa, because of its 1-dose schedule and its demonstrated immunogenicity and safety profile in children. To date, no studies of the field efficacy of any TCV have been conducted in Africa [30]. This protocol is designed to demonstrate the impact of Vi-TCV in an endemic setting in Malawi.

## METHODS

### Overview of the Trial

#### Efficacy Study

This study is a double-blind, individually randomized, controlled, efficacy trial with 2 vaccine groups: Vi-TCV and a meningococcal serogroup A conjugate vaccine (MCV-A). Participants (up to 28 000) will be randomized in a 1:1 ratio. Children 9 months through 12 years of age in the Blantyre area who meet the inclusion criteria will be eligible for enrollment. Immediate adverse events will be monitored for all participants. Serious adverse events (SAEs) in all participants will be monitored through to the end of the trial.

For the evaluation of efficacy, passive surveillance of febrile illnesses with blood culture collection will be conducted for 24–30 months for each individual, to identify typhoid fever cases among vaccinated subjects. Additional information, including the signs and symptoms of the illness and treatment given, will be collected from any child who has a blood culture obtained. Any child with blood culture–confirmed typhoid fever will have follow-up visits every other week until the illness resolves. Vaccine efficacy (VE) will be evaluated when the prespecified number of cases is reached, after a minimum of 2 years of follow-up on each participant.

The primary study hypothesis is that the Vi-TCV vaccine is efficacious in preventing typhoid fever, as confirmed by blood culture. We will test the 1-sided null hypothesis (H_0_: VE ≤ 0). The primary endpoint is clinical typhoid fever, confirmed by blood culture, with cases identified by passive surveillance in hospital and clinics. The secondary study hypothesis is that the safety profile of the Vi-TCV vaccine is adequate (Table 1).

**Table 1.** Study Objectives and Endpoints

**Primary objective**	1. To determine the efficacy of Vi-TCV in reducing rates of symptomatic, blood culture–confirmed *Salmonella* Typhi infection among children who receive Vi-TCV, compared to children who receive MCV-A.
**Secondary objectives**	1. To determine the safety profile of vaccination with Vi-TCV or MCV-A.
	2. To determine the immunogenicity of Vi-TCV in a subset of participants, by age group, as measured by serum, anti-Vi, IgG antibodies (percent seroconversion and geometric mean titer) at approximately 28 days following vaccination and at 2 years following vaccination.
	3. To determine the number of blood culture–confirmed cases of typhoid fever prevented during the study period, by comparing the incidence of blood culture–confirmed typhoid fever in participants receiving Vi-TCV to the incidence in participants receiving MCV-A.
**Exploratory objectives**	1. To determine the effect of Vi-TCV on the number and duration of hospitalizations due to blood culture–confirmed typhoid fever and the number of hospitalizations due to typhoid fever prevented by Vi-TCV.
	2. To determine the effect of Vi-TCV on the duration of hospitalization for febrile illness during the study period.
	3. To determine the effect of Vi-TCV for preventing all-cause hospitalizations and the number of hospitalizations prevented during the study period.
	4. To describe the clinical characteristics of blood culture–confirmed typhoid fever in study participants, including the percentage of participants with specific signs and symptoms.
	5. To determine the effect of Vi-TCV on outpatient visits for fever and the number of visits prevented during the study period.
	6. To determine the effect of Vi-TCV on the number of outpatient and hospitalized cases of clinically diagnosed typhoid fever
	7. To determine the effect of Vi-TCV on hospitalizations for febrile illness and the number of hospitalizations prevented during the study period.
	8. To determine the effect of Vi-TCV on antibiotic usage and the number of antibiotic courses and days of antibiotic use prevented during the study period.
	9. To determine the effect of Vi-TCV on all-cause mortality and the number of deaths prevented during the study period.
	10. To compare the number of episodes of illness for which blood cultures are collected during the study period between the Vi-TCV and MCV-A groups.
	11. To compare the incidence of hospitalizations for meningitis between the Vi-TCV and MCV-A groups.
	12. To evaluate the rate and recurrence of ileal perforations secondary to typhoid fever.
	13. To determine the effect of Vi-TCV on the incidence of complications of typhoid fever (eg, perforations, acute abdominal procedures, death) and the number of complications prevented during the study period.
	14. To determine the persistence of serum anti-Vi IgG antibodies in a subset of participants, by age group, at approximately 2 years following vaccination.
	15. To evaluate the efficacy and above outcomes by age groups.
	16. To compare the anti-measles IgG percent seroprotection and geometric mean concentrations among 9–11-month-old children receiving Vi-TCV and measles-rubella vaccine and children receiving MCV-A and measles-rubella vaccine.
	17. To evaluate the relationship between serum anti-Vi IgG at 28 days post-vaccination and the development of symptomatic, blood culture–confirmed *Salmonella* Typhi infection among children in the immunogenicity subset.

Abbreviations: IgG, immunoglobin G; MCV-A, a meningococcal serogroup A conjugate vaccine; TCV, typhoid conjugate vaccine.

#### Immunogenicity and Reactogenicity Sub-study

A subset of 600 children (200 in each of 3 age groups: 9–11 months, 1–5 years, and 6–12 years) will be included in an Immunogenicity and Reactogenicity Sub-study. More stringent exclusion criteria will apply for this subset. Serum specimens will be collected on Day 0 (before vaccination) and on post-vaccination Days 28 and 730 from all children included in the sub-study. For the children in the 9–11 month group, Vi-TCV or MCV-A will be administered with a measles-rubella–containing vaccine, as per the Malawi Expanded Programme on Immunization (EPI) schedule. These 9- to 11-month-old children will, additionally, have antibodies to measles and rubella assessed on Days 0 and 28. All children in the sub-study will be assessed at Days 3 and 7 following vaccination for the solicitation of local and systemic adverse events. Solicited and non-solicited non-SAEs will be assessed at Days 28 and 180. Serious adverse effects will be reported throughout the trial.

### Trial Experimental and Control Vaccines

#### Rationale for Study Vaccine

Vi-TCV (TypBar-TCV) is a single-dose vaccine licensed down to 6 months of age, making it feasible for incorporation into the routine EPI schedule. Vi-TCV is the only TCV to have been prequalified by the WHO [29].

#### Rationale for Control Vaccine

The MCV-A, MenAfriVac, is a single-dose vaccine licensed for use from 9 months of age; it is prequalified by WHO and provides protection against disease caused by group A *Neisseria Meningitidis*. While Malawi is outside the Africa meningitis belt, meningococcal A infection is a serious disease with high case-fatality rates and long-term sequelae among survivors, and so this vaccine is of some benefit to the children in this trial.

#### Study Population

Children aged 9 months through 12 years who reside within the townships of Ndirande and Zingwangwa, Blantyre, will be considered for enrollment.

#### Inclusion/Exclusion Criteria

The study will enroll healthy children between 9 months and 12 years/364 days of age at the time of study vaccination. Inclusion and exclusion criteria are broad for the main efficacy study, to simulate the general population of the Blantyre area (Table 2). The safety and immunogenicity sub-study will exclude children with known acute or chronic illnesses or severe malnutrition, which could interfere with the assessment of the safety and immunogenicity of the vaccine (Table 3).

**Table 2.** Inclusion and Exclusion Criteria

**Inclusion criteria**	A healthy male or female child between the ages of 9 months and 12 years/364 days at the time of study vaccination.
	A child whose parent or guardian resides primarily within the Ndirande or Zingwangwa study areas at the time of study vaccinations, and who intends to be present in the area for the duration of the trial.
	A child whose parent or guardian has voluntarily given informed consent.
**Exclusion criteria**	A history of documented hypersensitivity to any component of the vaccine.
	Prior receipt of any typhoid vaccine in the past 3 years.
	A history of a severe allergic reaction with generalized urticarial, angioedema, or anaphylaxis.
	Any condition determined by the investigator to be likely to interfere with evaluation of the vaccine, to be a significant potential health risk to the child, or to make it unlikely that the child would complete the study.
**Temporary exclusion criteria**	The following will be considered temporary contraindications to enrollment and vaccination. If these apply, the participant will be temporarily excluded for vaccination until 48 hours has passed. A reassessment will be needed to ensure these temporary exclusion criteria no longer exist.
	• Reported fever within 24 hours prior to vaccination. • Use of anti-pyretics within 4 hours prior to vaccination. • Receipt of measles-rubella vaccine in the 1 month prior to enrollment, as determined by parental history or vaccination card.

**Table 3.** Additional Exclusion Criteria for Immunogenicity and Reactogenicity Sub-study

• A known history of diabetes, tuberculosis, cancer, chronic kidney disease, heart disease, liver disease, a progressive neurological disorder, poorly controlled seizures, or a terminal illness.
• Severe malnutrition, as determined by mid-upper arm circumference < 12.5 cm for children younger than 5 years.
• The receipt of any other investigational intervention in the prior 6 months or the anticipated receipt during the course of the study.
• The receipt of blood products in the last 6 months.
• A known human immunodeficiency virus infection or exposure, or any other immunosuppressive condition.
• The receipt of systemic immunosuppressants or systemic corticosteroids.
• The receipt of any measles-rubella–containing vaccine for children younger than 1 year of age.

#### Recruitment and Vaccination

Recruitment will primarily occur in schools, with community and health-care centers also used. In schools, children will be given study information to take home and parents will be invited to attend a school-based vaccine clinic. The study will be explained, the requirements discussed, and questions answered by study staff. The parents/guardians of the children will then have the opportunity to provide informed consent. Assent will be obtained for all children 8 years of age and older. After informed consent and assent are obtained, each potential participant will be screened for eligibility according to the inclusion/exclusion criteria. The medical history, concomitant medications, and measles-rubella vaccination history, along with temperature, will be recorded. Once this is complete, and all eligibility criteria are verified, participants will be administered either Vi-TCV or MCV-A.

All study participants will be asked to remain in the study area for at least 30 minutes immediately following vaccination, in case there is a serious reaction. Any immediate reactions will be assessed and recorded.

### Randomization

Enrolled participants will be randomly assigned in a 1:1 ratio to either the Vi-TCV group or the MCV-A group, using block randomization, with varying block sizes from 6–12. The random allocation sequence will be generated by the blockrand package (version 1.3) in R (version 3.4.1) [31, 32].

Study participants, their family members, and the study staff involved in recruitment and eligibility will be unaware of the assigned vaccine group. The staff who prepare and administer the vaccines will be aware of the assigned vaccine group, but will not be involved in passive surveillance or any other subsequent study procedures.

An unblinded version of the allocation sequence will be maintained by an unblinded site statistician for use in unblinding according to protocol or for the assignment of replacement vials, should the original assigned vaccine be damaged or prepared incorrectly. Both the site statistician and the site data manager programming the study tablets have access to the unblinded randomization table, but will not be part of the clinical team and will not share the list with any team members responsible for study conduct.

This is a single-dose vaccine intervention and, therefore, we anticipate that any circumstances warranting unblinding will be rare. However, circumstances may arise where unblinding is needed prior to the end of the study. In such circumstances, the Independent Safety Monitor, who will be a physician in Malawi who is not a study investigator, will assess the child. The details will be reported to the Sponsor and the Data Safety Monitoring Board (DSMB). If necessary, the DSMB will recommend unblinding to the study sponsor. Any event of unblinding will be fully documented in the case report form.

### Follow-up

#### Passive Surveillance for Efficacy Study

**Figure 1.**
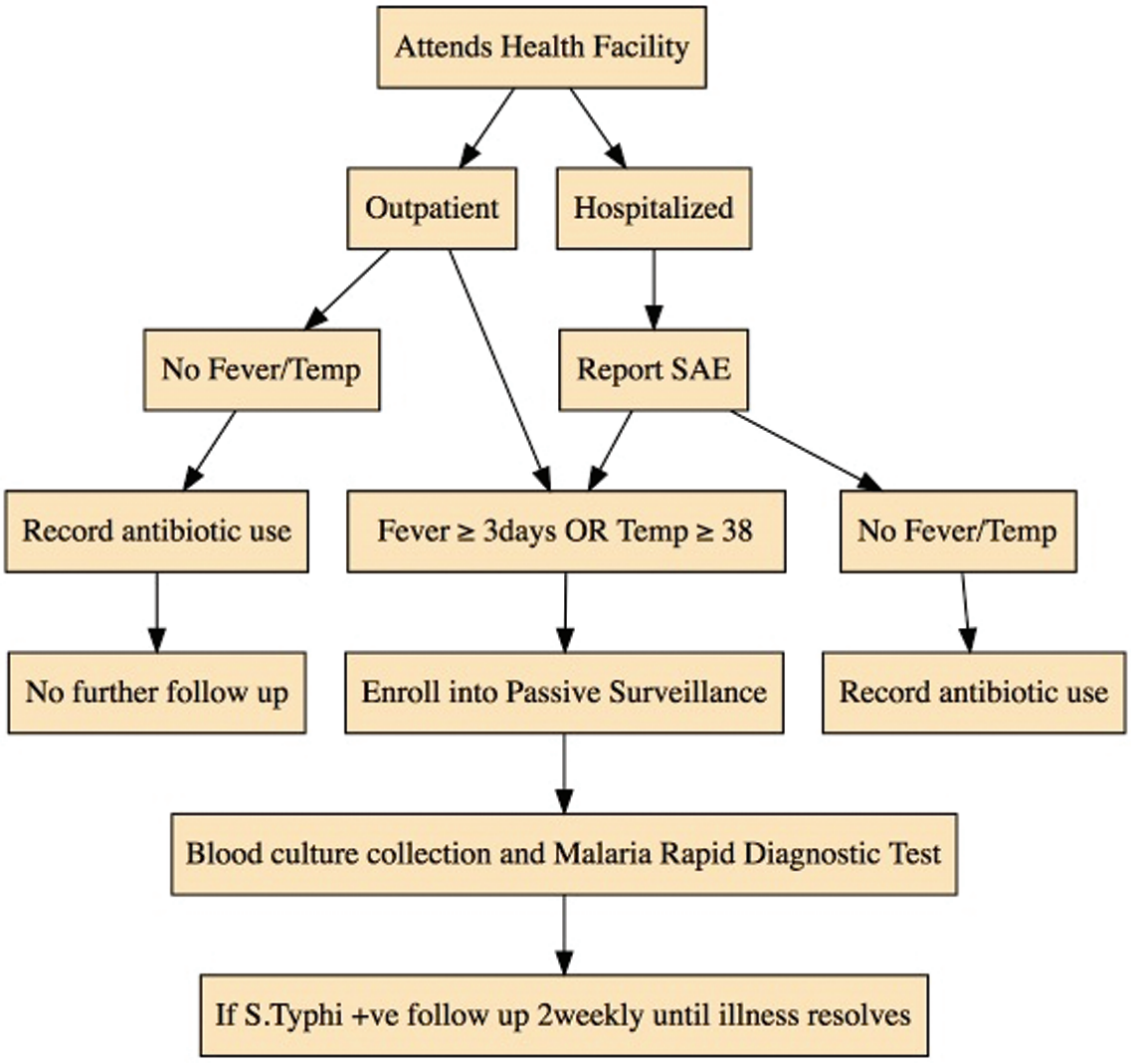
Passive surveillance enrollment flow. Abbreviations: SAE, serious adverse event; VE, vaccine efficacy.

Passive surveillance will be conducted throughout the study to identify outcomes among vaccinated subjects. All children attending primary or secondary health facilities in the study area will be screened, and those enrolled in the trial will have data pertinent to all endpoints collected. Those who meet the protocol-defined specimen collection criteria will have a blood culture collected (Figure 1). If a blood culture is positive for *S*. Typhi, the participant will be followed up by study staff to ensure appropriate treatment. Further information on blood-culture–confirmed typhoid illness will be obtained, and the participant will be followed every 2 weeks until asymptomatic.

To determine the effect of vaccination on the number of outpatient visits, number of hospitalizations, and antibiotic usage, passive surveillance will be conducted throughout the study for study participants who visit outpatient facilities or tertiary-level facilities or who are admitted to QECH but do not meet the protocol-defined specimen collection criteria. Information will be recorded, including the purpose of the visit, duration of symptoms, primary diagnosis, prescribed treatment, and dates of admission and discharge/loss to follow-up/death. SAEs, whether or not associated with study participation, will be recorded for the duration of the study.

### Active Follow-up for Sub-study

Children in the immunogenicity and reactogenicity sub-study will have home or clinic visits on Days 3, 7, and 180 following vaccination, at which solicited and unsolicited adverse events will be recorded. On Days 28 and 730 following vaccination, participants will attend the clinic for a 5 mL blood draw to measure typhoid-Vi antibody responses (all age groups) and measles and rubella antibody responses (9- to 11-month-old age group only. Children in the sub-study who are less than 5 years of age will have their height, weight, and mid–upper arm circumferences measured at baseline and at 2 years after vaccination.

### Statistics

#### Sample Size and Power Calculations

The number of blood-culture confirmed typhoid cases in the Vi-TCV and MCV-A groups are x_1_ and x_2_, respectively. The person-years of follow-up in the Vi-TCV and MCV-A groups are f1 and f2, respectively. The VE can be calculated as

$$\text{VE}=\left(1-\frac{x_{1}/f_{1}}{x_{2}/f_{2}}\right)\times 100\%=1-\frac{x_{1}}{x_{2}}h$$

where h = f_2_/f_1_ is the ratio of the follow-up person-years between the 2 vaccine groups, which is expected to be 1 due to randomization.

Now, letting P = x_1_/(x_1_+ x_2_), the proportion of blood culture–confirmed typhoid cases in the Vi-TCV group, we have

$$\text{VE}=1-\frac{P}{1-P}h$$

and

$$\text{P}=\frac{1-VE}{1-VE+h}$$

Then, we can test the null hypothesis that the vaccine has no protective efficacy (H_0_: VE ≤ 0) by testing the equivalent hypothesis that P ≥ 1(1 + h) = 0.5, since we expect h to be 1.

If we assume that the VE is 80%, then the hypotheses H_0_: VE ≤ 0 vs Ha: VE = 0.8 can be rewritten as H_0_: *P* ≥ .5 vs Ha: *P* = .16667. A sample size of 23 blood culture–confirmed typhoid cases is needed to achieve 90% power to detect the difference of 0.33 (= 0.5 − 0.16667) using a 2-sided exact test with a significance level of 0.025 (PASS 15). If we assume the attack rate is 0.0009 per year in the control group and each subject will be followed for 2 years, the total number of subjects that needs to be enrolled to result in the 23 required typhoid cases is 25 058, after accounting for a dropout rate of 15% (Table 4). With 28 000 children enrolled, the required number of cases is likely to be achieved, with an average of 24 months of follow-up per participant. Lower efficacy, fewer children enrolled, or a lower than assumed attack rate will require longer follow-ups to reach the minimum number of cases (see Tables 4 and 5 for sample size calculations for other parameter settings).

**Table 4.** Cases and Total Subjects Required for At Least 90% Power to Reject the Null Hypotheses of Vaccine Efficacy ≤ 0 or ≤ 30%, in 24 Months of Follow-up

	Null Hypothesis: VE ≤ 0%					Null Hypothesis: VE ≤ 30%				
	Proportion of Cases in Vaccinated Subjects					Proportion of Cases in Vaccinated Subjects				
VE Under Alternative Hypothesis	Under Null Hypothesis	Under Alternative Hypothesis	Number of Cases Required for 90% Power	Total Subjects (vaccine and control) Expected to Result in Required Number of Cases	Total Subjects, Allowing for 15% Loss to Follow-up	Under Null Hypothesis	Under Alternative Hypothesis	Number of Cases Required for 90% Power	Total Subjects (vaccine and control) Expected to Result in Required Number of Cases	Total Subjects, Allowing for 15% Loss to Follow-up
75%	0.5	0.20000	30	26 668	31 376	0.41176	0.20000	55	48 890	57 518
80%	0.5	0.16667	23	21 298	25 058	0.41176	0.16667	41	37 964	44 664
85%	0.5	0.13043	17	16 426	19 326	0.41176	0.13043	28	27 054	31830
90%	0.5	0.09091	15	15 152	17 826	0.41176	0.09091	22	22 224	26 146

These data are based on the assumptions that (1) there will be equal numbers of subjects and equal total follow-up times in the groups of subjects receiving the typhoid conjugate vaccine and the control treatment; (2) there will be an attack rate of 0.0009 per year in unvaccinated individuals; (3) the type error rate (α) will equal 2.5%, 1-sided; and (4) each subject will be followed for 24 months.

Abbreviation: VE, vaccine efficacy.

**Table 5.** Cases and Total Subjects Required for At Least 90% Power to Reject the Null Hypotheses of Vaccine Efficacy ≤ 0 or ≤ 30%, in 30 Months of Follow-up

	Null Hypothesis: VE ≤ 0%					Null Hypothesis: VE ≤ 30%				
	Proportion of Cases in Vaccinated Subjects					Proportion of Cases in Vaccinated Subjects				
VE Under Alternative Hypothesis	Under Null Hypothesis	Under Alternative Hypothesis	Number of Cases Required for 90% Power	Total Subjects (vaccine and control) Expected to Result in Required Number of Cases	Total Subjects, Allowing for 15% Loss to Follow-up	Under Null Hypothesis	Under Alternative Hypothesis	Number of Cases Required for 90% Power	Total Subjects (vaccine and control) Expected to Result in Required Number of Cases	Total Subjects, Allowing for 15% Loss to Follow-up
75%	0.5	0.20000	30	21 334	25 100	0.41176	0.20000	55	39 112	46 016
80%	0.5	0.16667	23	17 038	20 046	0.41176	0.16667	41	30 372	35 732
85%	0.5	0.13043	17	13 142	15 462	0.41176	0.13043	28	21 644	25 464
90%	0.5	0.09091	15	12 122	14 262	0.41176	0.09091	22	17 778	20 916

This data is based on the assumptions that (1) there will be equal numbers of subjects and equal total follow-up times in the groups of subjects receiving the typhoid conjugate vaccine and the control treatment; (2) there will be an attack rate of 0.0009 per year in unvaccinated individuals; (3) the type error rate (α) will equal 2.5%, 1-sided; and (4) each subject will be followed for 30 months.

Abbreviation: VE, vaccine efficacy.

### Statistical Analyses

In the primary analysis, subjects will be analyzed according to the vaccine received, and those subjects who are not vaccinated or who are provided no follow-up after vaccination will be excluded from the analysis. The estimated incidence rate of blood culture–confirmed typhoid will be calculated as the number of blood culture–confirmed typhoid cases, divided by the total number of person-years of follow-up. The incidence rates and corresponding 95% confidence intervals will be calculated for each vaccine group. The incidence rate ratio (IRR) will be calculated as the ratio of the incidence rate in the Vi-TCV group to that in the MCV-A group, and the VE will be calculated as (1-IRR) × 100%. Poisson regression, with an offset for the length of follow-up, will be used to compute the IRR, VE, and corresponding 95% confidence intervals, while adjusting for potential confounding variables (such as age and sex) as needed. A sensitivity analysis will be conducted, based on a per-protocol population that includes all vaccinated subjects and a follow-up time beginning 14 days after vaccination. Typhoid cases occurring before 14 days after vaccination will not be included in the sensitivity analysis of the per-protocol population. All analyses will be performed using Stata/SE (version 15).

#### Data Safety and Monitoring

An independent DSMB, including physicians and a statistician, will be established. This committee will meet before the study starts and will hold regular meetings for the duration of the trial. If a significant safety concern arises during the study, the DSMB Chair may convene a meeting to review safety and any other aspects of the study. Significant safety events may include, but are not limited to:

1. A death or life-threatening condition sustained by a participant, regardless of causality.
2. An unexpected, serious safety issue that is newly identified during the program and could expose participants to unnecessary risks.
3. Any other concern regarding participant safety, raised by any DSMB member, funder, investigator, or sponsor.

Proposed study amendments that significantly alter the treatment plan and/or deal with participant safety concerns will prompt an *ad hoc* meeting of the DSMB for review prior to the implementation of changes. This may require the suspension of enrollment pending a DSMB review.

### Ethical Considerations

#### Human Subjects’ Protections

The study will be conducted in accordance with the principles of the Declaration of Helsinki and in accordance with the principles of Good Clinical Practice (GCP).

#### Ethical/Institutional Review

This protocol, informed consent document, proposed advertising material, and participant information sheet will be submitted to the Malawi National Health Science Research Committee, the University of Maryland Institutional Review Board, and the University of Liverpool Research Ethics Committee for written approval.

#### Informed Consent

Individual written informed consent will be sought from each child’s parent or guardian, according to GCP. The consent form will be signed or a thumbprint provided by the child’s parent or guardian before any procedures are conducted. If a parent or guardian is unable to read or sign the consent form, an impartial witness will assist for the entire informed consent process, according to GCP. Written assent will be obtained from all children aged 8 and over.

### Data

#### Data Collection and Entry

Study data will be entered in real time into electronic tablets using the REDCap platform and uploaded daily by the in-country data team to a cloud-based, secure database. Individuals will be assigned personal identification numbers, readable from barcode or QR code stickers inserted in their individual health care records (which are held by the individual in Malawi) and used for consent and assent forms and for laboratory specimens. Daily quality assurance checks and the reconciliation of recruitment and other data will occur between the data teams at the site and the Sponsor. The Sponsor will oversee the overall data management and quality assurance procedures.

#### Quality Management

The study will be conducted in accordance with the protocol-specific quality management plan, designed in conjunction with the University of Maryland Center for Vaccine Development Office of Regulatory Affairs and Quality Management. Staff will be trained in and adhere to the standard operating procedures (SOPs), contained in the study manual of procedures. Study data collection forms are designed to guide staff study conduct. All study staff will attend and record mandatory training in GCP, Human Subject Protection, and all SOPs prior to participant enrollment, with regular updates as stipulated.

Site monitoring will be conducted to ensure that human subject protection procedures and study procedures, including study vaccine administration and clinical data and biological specimen collection, are of high quality and that the study is conducted in accordance with the protocol. Any protocol violations will be recorded and reported accordingly to all IRBs and regulatory bodies, and a corrective action plan will be agreed and enacted.

After data have been entered in the study database, they will be checked systematically by data management staff, according to a prespecified data validation plan agreed by the site and the Sponsor. All listings of the database will be reviewed and discussed for data quality and will be assessed for consistency and medical plausibility. After the resolution of all issues, the statistical analysis plan will be finalized and the database will be locked after the resolution of any remaining queries. A robust audit trail will be kept of all subsequent changes to the data.

### Plans for Dissemination of Results

When the clinical study report is completed, the investigators will share the summary results with the participating communities. It is anticipated that the results of this trial may have a significant bearing on policy decisions regarding the licensure and use of TCV in Malawi and other countries in Africa. Additionally, summary results will be provided to the ethics committees and regulators.

## DISCUSSION

This trial compares the effect of Vi-TCV to MCV-A on the blood culture–confirmed incidence of *S.* Typhi in Blantyre, Malawi, a typhoid-endemic setting. It is the first trial in Africa to test the effectiveness of TCVs and assess the safety and immunogenicity of this vaccine on the continent’s children.

The WHO has recommended the use of the vaccine from 6 months of age, with health economic modelling showing catch-up campaigns to be the most impactful and cost-effective, dependent on local epidemiology [33]. However, countries have many demands on their health-care dollars, and additional data will assist countries in deciding when and how to best employ this vaccine [29, 33]. The study will provide important data on a number of health outcomes beyond efficacy, including antimicrobial usage. The data for the 9-month-olds will inform introduction at that EPI visit, which could limit logistical and delivery costs and, in countries with high vaccine acceptability, like Malawi, could ensure high rates of uptake [34]. A recent Cochrane review affirmed the importance of these field trials [35]. This study will also demonstrate the effectiveness of the vaccine after a single dose, which could be useful to limit and control the spread of disease in the context of typhoid epidemics [12, 36].

## Notes

***Acknowledgments.*** The authors thank Dr Carol Tacket for her assistance in drafting the protocol.

***Financial support.*** This publication is based on research funded in part by a grant from the Bill & Melinda Gates Foundation (OPP1151153).

***Supplement sponsorship.*** This supplement is sponsored by the Center for Vaccine Development and Global Health (CVD) at the University of Maryland School of Medicine.

***Potential conflicts of interest.*** K. M. N. is a member of the World Health Organization Strategic Advisory Group of Experts on Immunization. All other authors report no conflicts. All authors have submitted the ICMJE Form for Disclosure of Potential Conflicts of Interest. Conflicts that the editors consider relevant to the content of the manuscript have been disclosed.
